# Supplementary material for: The interactome of intact mitochondria by cross-linking mass spectrometry provides evidence for coexisting respiratory supercomplexes
Source: Mol Cell Proteomics. 2017 Dec 8;17(2):216–32. doi: 10.1074/mcp.RA117.000470 (PMC5795388; doi:10.1074/mcp.RA117.000470)
Supplement: Supplemental Data [file supp_RA117.000470_133922_0_supp_23978_fzffwf.zip › spectra_annotation/mito_spectra_annotation/153-1-12-1-9-1.pdf]

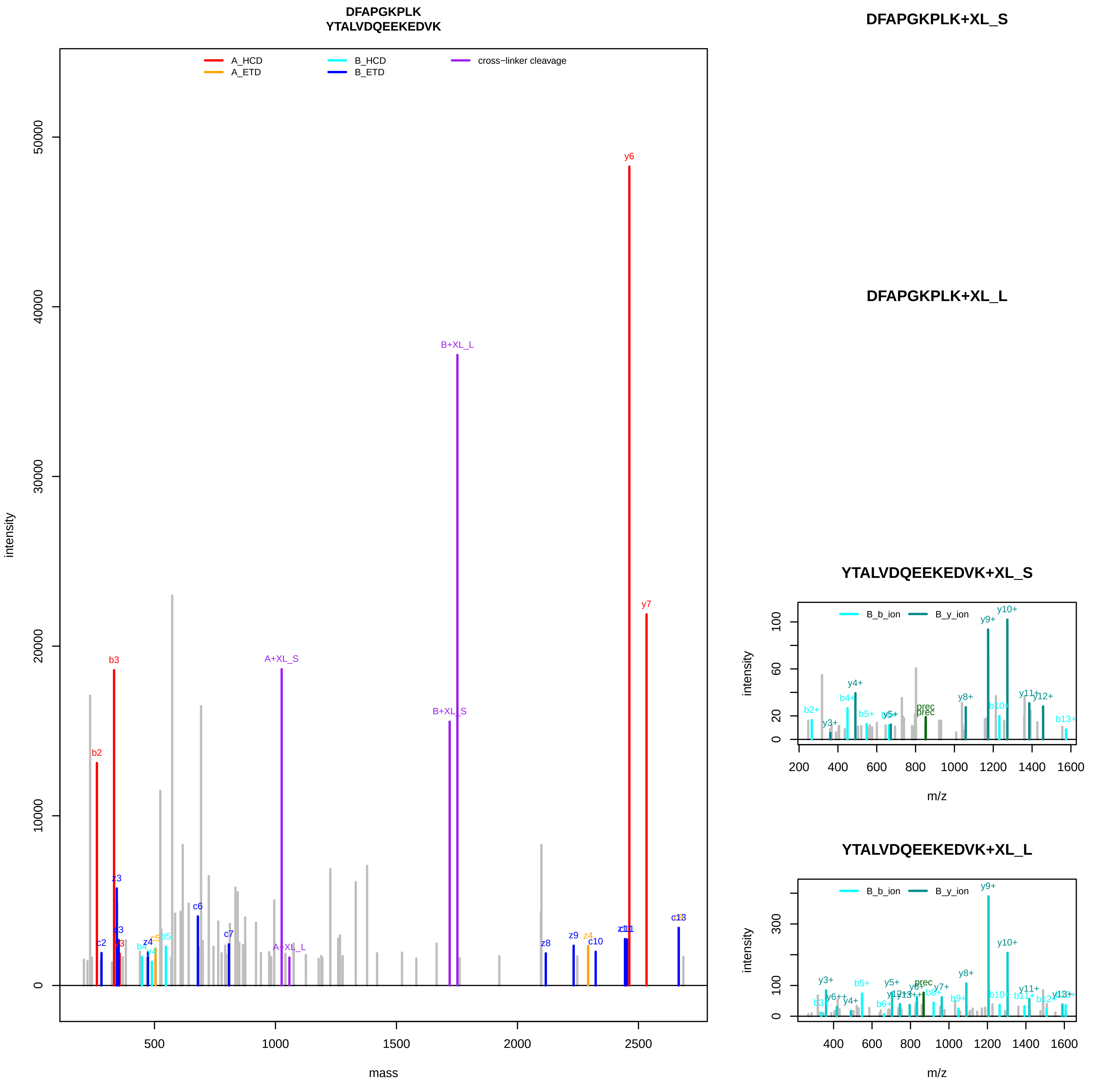

DFAPGKPLK
YTALVDQEEKEDVK
A_HCD
A_ETD
B_HCD
B_ETD
cross−linker cleavage
intensity
mass
0
10000
20000
30000
40000
50000
500
1000
1500
2000
2500
y6
B+XL_L
y7
b3
A+XL_S
B+XL_S
b2
z3
c6
c3
c2
y3
z4
c5
b5
b4
y4
c7
A+XL_L
z8
z9
z4
c10
c11
c13
DFAPGKPLK+XL_S
DFAPGKPLK+XL_L
YTALVDQEEKEDVK+XL_S
B_b_ion
B_y_ion
m/z
200
400
600
800
1000
1200
1400
1600
0
20
60
100
y10+
y9+
y4+
b4+
b2+
y3+
b5+
y5+
prec
y8+
b10+
y11+
y12+
b13+
YTALVDQEEKEDVK+XL_L
y9+
y10+
y3+
b5+
y5+
y6++
y4+
y8+
y7+
b3+
b6+
b8+
b9+
b10+
y11+
b11+
b12+
y13+
300
